# Assessment of Risk Factors for Rupture in Breast Reconstruction Patients with Macrotextured Breast Implants

**DOI:** 10.1007/s00266-022-03118-9

**Published:** 2022-10-13

**Authors:** Guido Paolini, Guido Firmani, Francesca Briganti, Mattia Macino, Simone Nigrelli, Michail Sorotos, Fabio Santanelli di Pompeo

**Affiliations:** 1 NESMOS (Neurosciences, Mental Health, and Sensory Organs) Department, Faculty of Medicine and Psychology, Azienda Ospedaliera Sant’Andrea – U.O.D. Chirurgia Plastica, Sapienza University of Rome, Via di Grottarossa 1035-1039, 00189 Rome, Italy; 2 Faculty of Medicine and Psychology, Sant’Andrea Hospital, Sapienza University of Rome, Via di Grottarossa 1035-1039, 00189 Rome, Italy; 3 Department of Biomedicine and Prevention, Tor Vergata University, Via Montpellier, 1, 00133 Rome, Italy; 4 NESMOS Department, Faculty of Medicine and Psychology, Chair of Plastic Surgery - Sant’Andrea Hospital, Sapienza University of Rome, Via di Grottarossa 1035-1039, 00189 Rome, Italy

**Keywords:** Breast implants, Macrotextured, Breast reconstruction, Implant rupture, Rupture risk factors

## Abstract

**Background:**

Breast implants (BI) are widely used in plastic surgery, though they are not lifetime devices. Average life before rupture is reported to be around 10–15 years. No consensus exists regarding which factors are involved.

**Objectives:**

Following FDA recommendations, this study aims at identifying potential risk factors by evaluating their effect on BI rupture cases.

**Methods:**

In this observational study, 763 BI patients were operated between 2003 and 2019, with a mean implant indwelling of 12.2 years. Patients that returned for follow-up were administered a questionnaire regarding postoperative lifestyle and habits. Implant rupture rate was 15.1%, while BI lifespan was 10.1 years. We obtained complete data from 191 breast implant patients (288 implants). Twenty-three potential risk factors were evaluated and divided in four categories: patient-related, surgery-related, postoperative complications/symptoms, and postoperative care/lifestyle habits. Odds Ratio (OR) for each factor was calculated. Linear regression analysis was calculated for those with a significant OR.

**Results:**

We report 120 patients (195 implants) with intact and 71 (93 implants) with ruptured devices. BIs were macrotextured in 95.1% of cases (86.8% Allergan BIOCELL). OR was significant for underwire bra use (OR: 2.708), car seat belts (OR: 3.066), mammographic imaging (OR: 2.196), weightlifting (OR: 0.407) and carry-on heavy purses and backpacks (OR: 0.347).

**Conclusion:**

Wearing underwire bras, seat belts and undergoing mammography increases the risk of rupture. Weightlifting and carry heavy bags do not increase that risk. Implant rupture is directly linked with time of indwelling. Postoperative recommendations in BI patients should consider findings from our study, though larger multicenter studies should be encouraged.

**Level of Evidence IV:**

This journal requires that authors assign a level of evidence to each article. For a full description of these Evidence-Based Medicine ratings, please refer to the Table of Contents or the online Instructions to Authors www.springer.com/00266.

**Supplementary Information:**

The online version contains supplementary material available at 10.1007/s00266-022-03118-9.

## Introduction

Breast implants (BIs) are class III medical devices according to the Food and Drug Administration (FDA) [1], a category reserved for high-risk devices which may cause illness or injury [2]. Despite this fact, recent estimates suggest that 3% of female adults have BIs [3]. Their popularity is owed to breast augmentations being among the top five most frequent cosmetic surgeries, while implant-based techniques represent the most commonly performed reconstructive procedures [4].

Industrial and clinical research have been conducted to improve upon BI characteristics, with the goal of reducing complications and enhancing aesthetic outcomes [5, 6]. Despite these efforts, the FDA cautions that BIs are not lifetime devices [7]. Some complications leading to removal or replacement are related to surgical technique (i.e., bleeding/hematoma, early seroma, infection), thus they are potentially predictable and possibly avoidable [8]. Implant rupture, which represents one cause for revisional surgery [9], is unpredictable, as it is unclear whether it depends on device failure or whether certain risk factors increase its likelihood. In fact, little is known regarding which factors can negatively affect BI survival, other than longevity of device indwelling.

Long-term observations from our prospectively-maintained breast surgery database highlighted a high number of BI rupture cases, which compelled us to identify which factors were associated with a higher likelihood of rupture, in accordance with the FDA’s “Guidance for Industry and Staff” on BIs [10]. Potential risk factors regarding patient characteristics, surgery, complications, postoperative care and lifestyle habits were assessed to verify their impact on rupture.

## Materials and methods

This retrospective observational study was conducted in accordance with the World Medical Association’s Declaration of Helsinki for ethical principles in medical research involving human subjects [11]. A prospectively-maintained breast surgery database was used to determine BI rupture rate, and calculate mean indwelling and lifespan. BI lifespan was calculated for patients with ruptured devices and was defined as the period of time between placement and rupture. BI indwelling was calculated for the overall population, by averaging the time between implantation and latest follow-up in patients with intact BIs, and BI lifespan in patients with ruptured devices.

The primary outcome was to determine which variables were associated with BI rupture [10]. These variables were subdivided into four categories: patient-related factors, surgery-related factors, postoperative complications/symptoms, and postoperative care/lifestyle habits. A total of 23 potential risk factors were identified and are further subdivided in Table 1.

**Table 1:** Identified risk factors for breast implant rupture, subdivided into four categories

Patient-related risk factors
Age Body mass index (BMI) Handedness (right-handedness, left-handedness or ambidexterity) Time between implantation and removal or exchange for rupture
Surgery-related factors
Implant manufacturer Implant degree of texturization Implant size (in cc) Indication for placement (reconstructive vs cosmetic procedure) Type of breast reconstruction procedure (only for reconstruction) Submuscular vs subglandular pocket (only for cosmetic procedure) Adjunct surgical procedures (i.e., autologous fat transfer, nipple-areola complex reconstruction, scar revisions) Pre- or postoperative radiotherapy
Postoperative complications
Capsular contracture (according to Baker’s classification, range: soft [I], minimal [II], moderate [III] and severe [IV]) History of trauma of the chest region following surgery
Postoperative care and lifestyle habits
Using an underwire bra Wearing a car seat belt Sleeping in a prone position Implant manipulations or massages following surgery Physical activity and sports Weightlifting (i.e., lifting weights heavier than 5 kg using arms on a daily basis) Use of heavy purses or backpacks Type of housework (subdivided, according to the frequency and duration of household chores, into: none, light, moderate and heavy) History of mammographic imaging following surgery, with Eklund-modified compression technique

### Patient population

In our database, we identified 763 patients who received BI placement for reconstructive or aesthetic purposes at our institution between 2003 and 2019, with a follow-up of at least 3 years. The database was used to retrieve surgery-related factors and postoperative complications. All patients were sent via email a questionnaire regarding postoperative care and lifestyle habits [Supplemental Material 1]. This study was conducted between June 2021 and January 2022. Patients who responded to the questionnaire were included, while those without recent diagnostic breast imaging according to FDA recommendations [12], and/or who did not respond were excluded.

### Statistical analysis

All variables were subdivided into two categories: dichotomous and continuous (Table 2). Time of implant indwelling was considered a continuous variable expressed in 1-month increments. We initially performed descriptive statistic calculations for continuous variables, assessing the average value, mode, median value and standard deviations. Nominal and ordinal variables were reported adding proportions and percentages. Pearson correlation coefficient was used for the following continuous variables: patient BMI, breast implant weight, time of implant indwelling and the dependent dichotomous variable implant integrity status (ruptured vs intact). In regard to the continuous variable “time of implant indwelling,” we compared averages between cases with BI rupture and cases with intact BIs. Odds Ratio (OR) for each factor was calculated for all dichotomous variables. Association between rupture and potential risk factors was evaluated by multivariate analysis. A contingency table was prepared, linking breast implant integrity status with type of implant-based surgery, and degree of capsular contracture, then with all dichotomous variables in our database. Later, we separately analyzed whether breast pain was correlated with BI rupture using Pearson correlation coefficient.

**Table 2:** Dichotomous vs continuous variable subdivision

Dichotomous variables	Continuous variables
Adjunct surgical procedures (i.e., autologous fat grafting) Capsular contracture Handedness History of trauma of the breast region History of mammographic imaging History of radiotherapy Breast implant integrity status (ruptured vs. intact) Implant manipulations or massages Physical activity and sports Sleeping in prone position Use of underwire bra Use of car seatbelt Weightlifting and use of heavy bags	Body mass index (BMI) Breast implant weight Time of implant indwelling Type of housework

We used OR values to determine the association between BI rupture rate and all variables from the four selected categories. An OR equal to or above 1 was considered significant. Finally, logistic regression was used to identify independent predictors of implant rupture. All variables were considered statistically significant for *p* values less than 0.05, with a confidence interval of 95%. Hosmer–Lemeshow test was performed to assess goodness of fit for logistic regression model. All statistical analyses were performed using SPSS^®^ software vers. 28.0 (IBM^®^ Corporation; Armonk, New York).

## Results

**Fig. 1:**
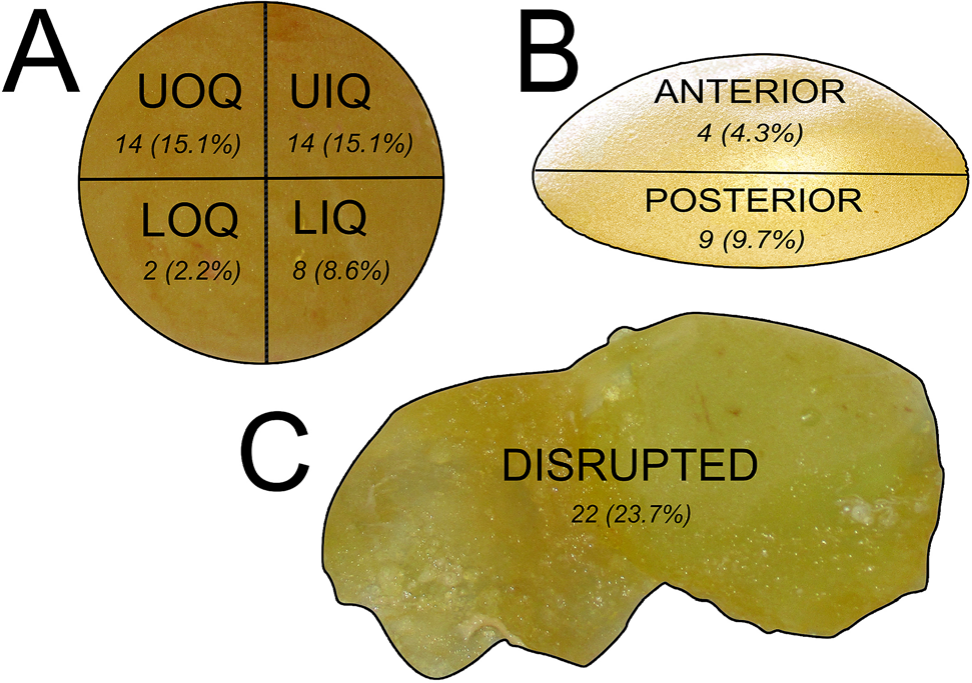
Breast implant (BI) rupture location and frequency. Rupture occurred either in one of the four possible quadrants of the BI (**A**), which are the (Upper Inner Quadrant [UIQ], Lower Inner Quadrant [LIQ], Lower Outer Quadrant [LOQ], Upper Outer Quadrant [UOQ]); in the anterior or posterior aspect with extensive damage (**B**); or caused complete disruption of the silicone elastomer (**C**)

The starting population of 763 patients had a mean age of 48.9 years and consisted of 580 primary (76%) and 183 revisional cases (24%). Mean BI indwelling was of 12.2 years (range: 3.2–30.5). One-hundred-fifteen BI rupture patients were identified (15.1%). Mean BI lifespan was of 10.1 years (range: 0.6–29.4). One-hundred-ninety-one (25.0%) patients responded to the questionnaire between June 2021 and January 2022, for a total of 288 breasts. The population included 254 (88.1%) breast reconstructions following mastectomy and 34 (11.9%) non-oncologic cases (including cosmetic augmentations and corrections of congenital breast deformities). Placement was submuscular in 255 (88.5%) and subglandular in 33 cases (11.5%). We report that 250 (86.8%) BIs were manufactured by Allergan (Allergan Inc., Irvine, CA, USA), and that overall 95.1% of all analyzed devices had a macrotextured outer surface. Additionally, 253 (87.8%) implants were shaped, while 35 (12.2%) were round. Patient demographics, surgery and BI characteristics are detailed in Table 3. The study included 93 (32.3%) BI ruptures: 84 had been placed for breast reconstruction following mastectomy, four during cosmetic breast augmentation and five for correction of congenital breast deformities. In 12 patients, rupture of the implant was bilateral, while it was unilateral in 69. Capsular contracture was grade I–II in 235 (81.6%) and grade III-IV in 53 patients (18.4%) (Table 4). Rupture was defined according to preoperative Magnetic Resonance Imaging findings as intracapsular when the periprosthetic capsule was intact despite a breach of the implant shell, which occurred in 69 instances (74.2%). Extracapsular rupture was defined as the event in which silicone extravasation occurred, which represents 24 cases (25.8%). Location of BI rupture was detected during revisional surgery and accurately described in 73 surgical reports, but was unknown in 20 (21.5%). Rupture was located in one of four quadrants. If two or more quadrants were affected, the implant was divided in an anterior and posterior aspect. If three or more quadrants were affected, the implant was considered completely disrupted. Frequency of BI rupture location is depicted in Figure 1, but was not deemed statistically significant (Figure 1).

**Table 3:** Demographic, surgery and breast implant characteristics of the patient population that responded to the questionnaire

Patient characteristics	Value (percentage) [ranges]
Number of patients	191
Number of BIs	288 (100%)
Mean age	49.2 years [19–81]
Mean weight	61.4 kg [41–93]
Mean height	1.63 m [1.50–1.87]
Mean BMI	23.2 kg/m^2^ [14.9–36.3]
Median year of implantation	2007 [2003–2019]
Median year of explantation/replacement	2014 [2011–2022]
Implantation at our institution:	
Primary surgery	244 (84.7%)
Revisional surgery	44 (15.3%)
Laterality of BI placement:	
Bilateral placement	198 implants (68.8%)
Unilateral placement	90 implants (31.2%)
Right side	46 (51.1%)
Left side	44 (48.9%)
Indications for BI placement:	
Unilateral breast cancer	183 (63.5%)
Bilateral breast cancer	58 (20.1%)
Prophylactic mastectomy	13 (4.5%)
Cosmetic breast augmentation	19 (6.6%)
Correction of congenital breast deformity	15 (5.2%)
Device placement:	
Submuscular	255 (88.5%)
Subglandular	33 (11.5%)
Breast reconstruction timing:	
Immediate	135 (53.1%)
Delayed	119 (46.9%)
Type of breast reconstruction:	
Implant-enhanced latissimus dorsi	137 (53.9%)
Tissue expander/implant exchange	19 (7.5%)
Wise pattern direct implant	66 (26.0%)
ADM assisted subpectoral	32 (12.6%)
Breast implant manufacturers:	
Third generation BIs or older	4 (1.4%)
Allergan Inc.	250 (86.8%)
GC aesthetics/eurosilicone	10 (3.5%)
Mentor worldwide LLC	7 (2.4%)
Establishment labs	8 (2.8%)
Sientra/silimed	7 (2.4%)
Unknown	2 (0.7%)
Breast implant shape:	
Anatomical	253 (87.8%)
Round	35 (12.2%)
Breast implant outer texture:	
Macrotextured	274 (95.1%)
Microtextured	0 (0%)
Smooth	12 (4.2%)
Unknown	2 (0.7%)
Mean BI size	351.8 cc [125–600]
BI integrity status:	
Intact	195 (67.7%)
Ruptured	93 (32.3%)
Intracapsular rupture	69 (74.2%)
Extracapsular rupture	24 (25.8%)
Indication of ruptured implants	
Breast reconstruction following mastectomy (unilateral, bilateral and prophylactic)	84 out of 254 (33.1%)
Cosmetic breast augmentation	4 out of 19 (21.5%)
Correction of congenital breast deformity	5 out of 15 (33.3%)
Laterality of patients with intact breast implants	110 (57.6%)
Bilateral	71 (64.5%)
Unilateral	39 (35.5%)
Right	22 (56.4%)
Left	17 (43.6%)
Laterality of patients with ruptured breast implants	81 (42.4 %)
Bilateral rupture	12 (14.8%)
Unilateral rupture	69 (85.2%)
Right	28 (40.5%)
Left	41 (59.4%)

**Table 4:** Postoperative care and lifestyle habits of the patient population

Patient characteristics	Value (percentage) [ranges]
Capsular contracture	
Baker I–II	235 (81.6)
Baker III–IV	53 (18.4)
History of trauma	Yes: 51 (17.7)
	No: 237 (82.3)
Use of underwire bra	Yes: 45 (15.6)
	No: 243 (84.4)
Wear car seat belts	Yes: 260 (90.3)
	No: 28 (9.7)
Prone sleep	Yes: 91 (31.6)
	No: 197 (68.4)
Implant manipulations or massages	Yes: 20 (6.9)
	No: 268 (93.1)
Physical activity and sports	Yes: 139 (48.3)
	No: 149 (51.7)
Weightlifting	Yes: 181 (62.8)
	No: 107 (37.2)
Type of housework	None: 17 (5.9)
	Mild: 196 (68.1)
	Moderate: 36 (12.5)
	Heavy: 39 (13.5)
Use heavy purses and backpacks	Yes: 182 (63.2)
	No: 106 (36.8)
Mammography imaging	Yes: 136 (47.2)
	No: 152 (52.8)

**Fig. 2:**
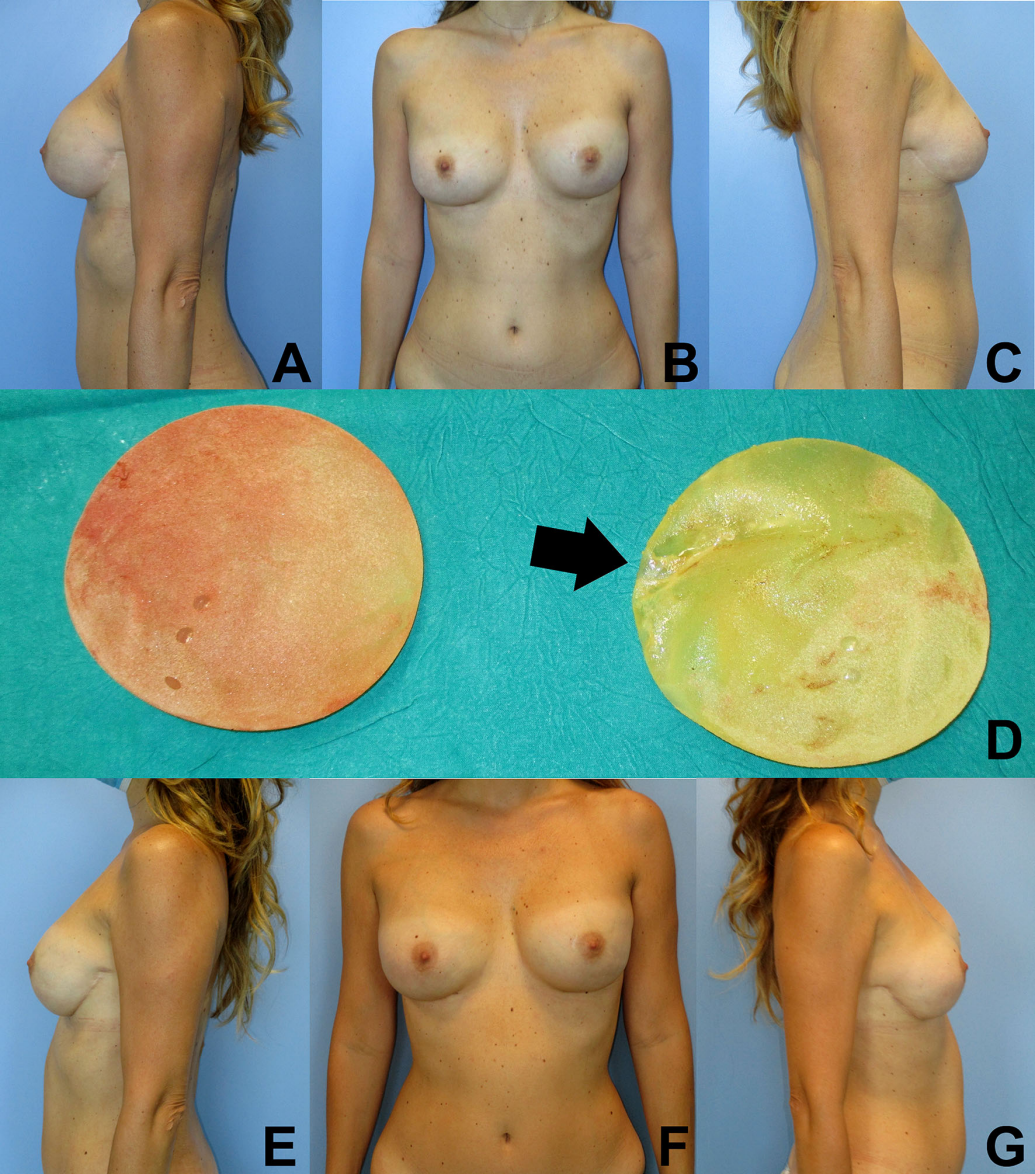
Patient with bilateral implant-enhanced latissimus dorsi breast reconstruction with left implant rupture, in the frontal (**B**) and lateral (**A** and **C**) views. Intraoperative assessment of breast implants, showing a ruptured left implant in the UIQ (**D**). Postoperative photographs (**E**, **F **and **G**) are displayed at 24 months from bilateral breast implant replacement surgery

**Fig. 3:**
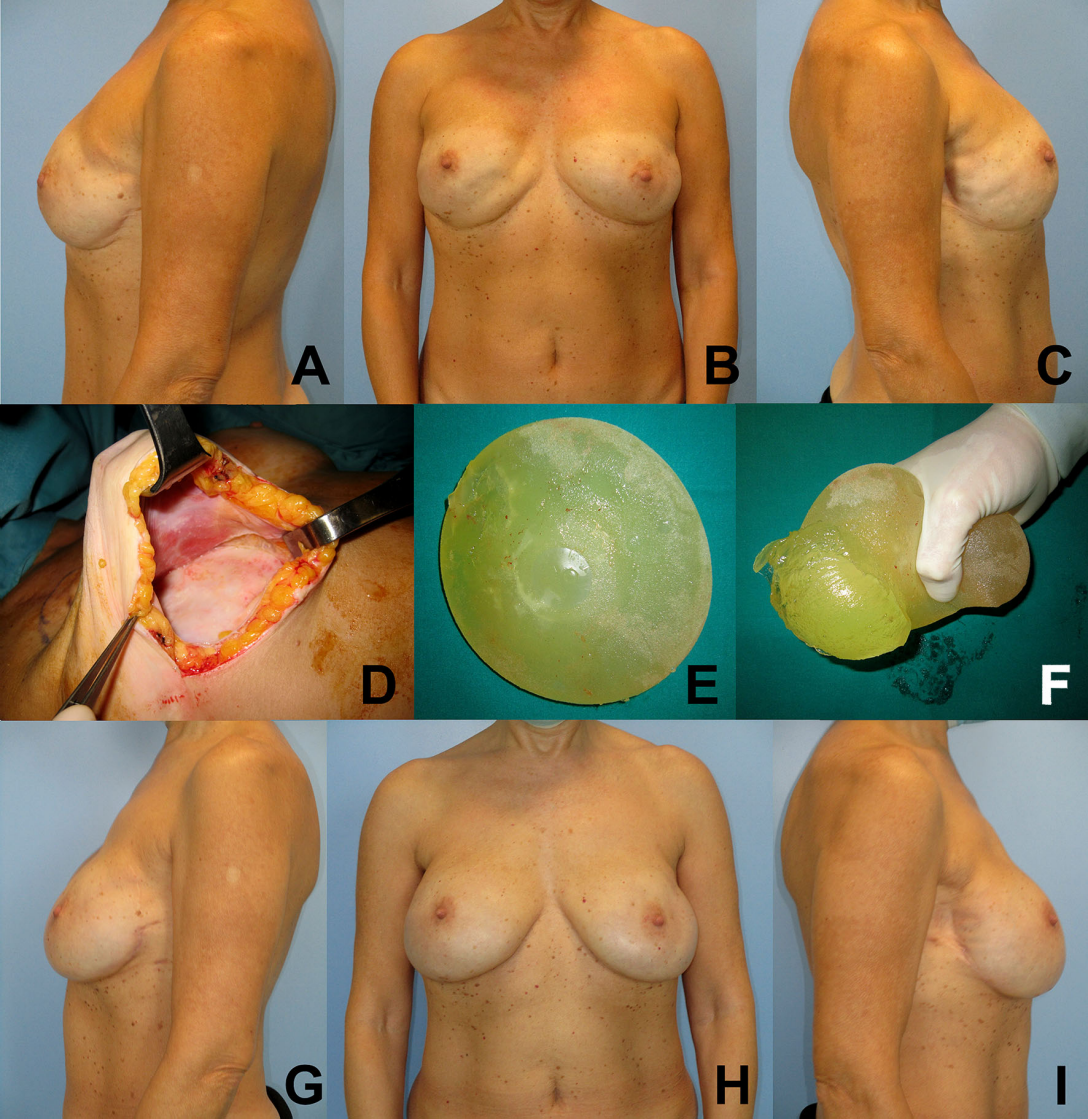
Patient with bilateral submuscular direct-to-implant breast reconstruction with right implant rupture, in the frontal (**B**) and lateral (**A** and **C**) views. Intraoperative assessment of breast implants, showing a ruptured right implant in the posterior aspect (**D**, **E** and **F**). Postoperative photographs are displayed at 12 months from bilateral breast implant replacement surgery

In terms of postoperative care and lifestyle habits, the use of underwire bras [OR: 2.708 (1.242–5.903), 95% CI, *p* = 0.012], wear car seat belts [OR: 3.066 (1.959–9.805) 95% CI, *p* = 0.049] and prior mammographic imaging [OR: 2.196 (1.252–3.853), 95% CI, *p* = 0.006] increased risk for BI rupture; while weightlifting [OR: 0.407 (0.319–0.754), 95% CI, *p* = 0.004] and use of heavy purses or backpacks [OR: 0.347 (0.193–0.625), 95% CI, *p* < 0.001] did not increase BI rupture rate. All other factors, including prone sleep (*p* = 0.403), history of fall or trauma of the chest region (*p* = 110), physical activity (*p* = 0.114), implant manipulations (*p*) and radiotherapy (*p* = 0.806), were not deemed statistically significant (Table 5). Regarding patient-related risk factors for implant rupture, longer implant indwelling was statistically significant [OR: 1.040 (0.999–1,009), 95% confidence interval (CI), *p* = 0.015], while age, BMI and handedness were not. No surgery-related risk factor was statistically significant, including indication for surgery and adjunct surgical procedures (i.e., fat grafting, nipple reconstruction, balancing procedures). Among postoperative complications, capsular contracture and history of trauma were not significant (Figures 2 and 3).

**Table 5:** Multivariate analysis: significant factors associated with breast implant rupture

Parameter	Odds ratio	95% confidence limits		*p* value
		Lower	Upper	
Time	1.040	0.999	1.009	0.015
Underwire bras	2.708	1.242	5.903	0.012
Car seat belts	3.066	1.959	9.805	0.049
Mammographic imaging	2.196	1.252	3.853	0.006
Weightlifting	0.407	0.319	0.754	0.004
Heavy purses and backpacks	0.347	0.193	0.625	< 0.001

## Discussion

Device rupture is considered one of the primary safety concerns presented by gel-filled BIs [10]. Despite great technological advancements achieved in the last decades, these devices still cannot be expected to last a lifetime and there is no agreement on their lifespan [7], though most surgeons will estimate it to be around 10–15 years [13, 14]. Nowadays, BI manufacturers recognize rupture as a potential complication [15].It is also fairly accepted that the longer the indwelling, the likelier it is for patients to experience adverse outcomes requiring surgery [16]. To strengthen BI safety, the FDA proposed black box warning that includes requesting manufacturers to improve labelling and patient information, outlining implant rupture as a relevant concern [17], as well as updating rupture screening recommendations [18]. Other means include pushing for improved reporting strategy of confirmed rupture cases, and conducting Premarket Approval (PMA) Core studies. Some Plastic Surgery societies have been proactive regarding the reporting of rupture cases. In fact, the European Association of Plastic Surgeons (EURAPS) was requested in 2018 by the European Commission to provide their expert opinion concerning the Device Specific Vigilance Guidance (DSVG) on BIs. The EURAPS stated that events such as rupture are well-known and highly clinically significant incidents, thus proposed them as appropriate to be reported as individual cases, rather than using Manufacturers’ Periodic Summary or Trend Reports [19]. However, individual reporting is a precious source of knowledge that will collectively take time to produce viable information. On the other hand, core studies are characterized by relatively limited sample sizes, thus we believe that they may not fully address all our concerns regarding device rupture. For this reason, the FDA advocates using other sources of information, such as retrospective or prospective studies to garner long-term rupture rate data [10].

On a related subject, there are staggering inconsistencies in literature regarding terminology and the definition of “BI lifespan.” Some authors consider it as the time between implantation and removal or replacement [14], while others define it as the time between implantation and rupture [14]. We chose the latter definition in our study. Terminology is key since implant rupture accounts for several cases of removal or exchange, but remains highly unpredictable [20]. Core studies report rupture rates of BIs produced by various manufacturers at 10 years follow-up, which range from 4.9 to 24.2% in cosmetic augmentations, and 9.8–35.4% in reconstruction cases [21–27]. This data is summarized in Tables 5 and 6.

**Table 6:** Comparison of breast implant rupture rates across primary augmentation, revision augmentation, primary reconstruction and revision reconstruction patients

						Primary augmentation	Revision augmentation	Primary reconstruction	Revision reconstruction
	Tested implant	Manufacturer	Implant shape	Total population	Follow-up	Breast implant rupture rates			
Caplin et al. [24]	MemoryGel	Mentor	Round	1008	10 years	24.2%	23.7%	32.7%	38.7%
Hammond et al. [25]	MemoryShape (contour profile gel)	Mentor	Shaped	955	10 years	6.6%	9.6%	18.9%	0%
El-Haddad et al. [27]	Sebbin silicone textured and smooth	Sebbin	Round	205	10 years	7.4%		21.2%	
Duteille et al. [28]	Cristalline paragel	Eurosilicone S.A.S. (GC aesthetics)	Round and anatomical	995	10 years	4.9%	2.3%	0%	0%
Spear et al. [22]	Natrelle round	Allergan	Round	715	10 years	9.3%	5.4%	35.4%	
Maxwell et al. [23]	Natrelle style 410	Allergan	Anatomical	941	10 years	17.7%	14.7%	12.4%	19.6%
Stevens et al. [26]	High-strength cohesive (HSC), high-strength cohesive plus (HSC+)	Sientra	Round and shaped	1788	10 years	7.8%	5.2%	9.8%	–

Data were retrieved from industry-funded core studies. “–“ is used when data in not available. The terms “Anatomical” and “Shaped” are used interchangeably throughout the table

BI rupture rate is defined as the ratio between ruptured implants (numerator) over the total number of implanted individuals (denominator). Obtaining a realistic account of rupture rate that is not limited by industry-sponsored studies is particularly challenging for three reasons. Firstly, data provided by manufacturers might overestimate implant longevity due to the nature of laboratory stress tests performed on BIs [28]. These stress tests are limited in their ability to reproduce in-vivo conditions of implant indwelling, meaning that they are not necessarily predictive of device failure [10]. Secondly, the number of women with BI in each country (i.e., the denominator) is not known, since National Implant Registries are either inexistent, not operational or not currently efficient [29, 30]. Thirdly, most BI ruptures are silent and clinically undetectable [31], which makes the numerator difficult to assess. In fact, some ruptures may come across as incidental findings from revisional surgeries performed for other reasons or diagnostic images requested for other health concerns [32]. It is worth nothing that 25.8% of all ruptures were extracapsular. Extracapsular rupture can sometimes represent the progression of an intracapsular one, with additional silicone seepage occurring over time [16, 33]. We speculate that BI ruptures likely occurred earlier than when radiologically detected.

Some authors proposed recommendations for optimal pre- and postoperative care of textured implants [34], or breast augmentations in general [35]. Nevertheless, standards of care are still elusive and many of the still used recommendations are not evidence-based, as they have not been proven to reduce incidence of rupture. Questions remain whether this event is a device-related complication or whether it may be influenced by certain factors. The most notable study addressing this topic was conducted by Feng et al. [36] in 1999. They analyzed potential risk factors in a population of 842 patients who received implant removal between 1990 and 1996. The authors found that the following factors were associated with a significantly higher risk of rupture rate: increasing age of implant, retroglandular placement, severe capsular contracture (Baker’s grades III and IV), and presence of local symptoms. In our study, 23 variables were selected, in accordance with the FDA’s recommendations regarding demographics, baseline characteristics and complications for patients in study cohorts [10]. Similarly to Feng et al.’s study, we found longer implant indwelling to increase rupture rate. However, his population differs significantly from our own, as 69.5% of their patients had undergone a cosmetic procedure, 28.2% received a reconstruction following breast cancer and 2.3% corrected a congenital deformity. Additionally, they reported that 67.4% received a subglandular placement while 32.6% had their BIs placed submuscularly. In our study, 88.1% of patients received a reconstruction following mastectomy, while 6.6% a cosmetic breast augmentation and 5.2% a congenital deformity correction. Implant pocket was submuscular in 88.5% of cases, and subglandular in 11.5%. Subglandular placement was not statistically significant in our study. The role of submuscular pockets in BI rupture is a controversial topic. Though Feng et al. found it to be protective compared to subglandular placements, Hadad et al. [37] found it to be a significant risk factor in their retrospective cohort study on 362 breast augmentation patients. Conversely, Hölmich et al. [38] concluded in their observational study on 533 cosmetic patients that no significant association was found with the position (subglandular or submuscular) of the implant. Additionally, despite featuring several implant-based reconstruction techniques in our study (submuscular direct-to-implant, implant-enhanced latissimus dorsi, tissue expander/implant exchange) we did not find submuscular placement to affect rupture rate in a statistically significant manner. Regarding the location of rupture, we found that most occurred in the upper quadrants. This is peculiar since the most exposed area and thus theoretically more at risk of rupture in cosmetic augmentations is the lower outer quadrant. This might be a result of the fact that our cohort mostly features reconstructive cases.

Our study, though limited by a small patient population, is focused on reconstructive surgery and features a long follow-up period. Furthermore, it was able to clarify the role of patient-related factors and lifestyle habits, which are poorly investigated in the literature. We found the use of underwire bras, car seat belts and prior mammographic screening to increase implant rupture rate. Avoidance of bras with an underwire in the postoperative period are a common recommendation for implant-based breast reconstructions [39], as they could impair healing of an inferior incision [40]. We inquired our patients on long-term use of underwire bras after surgery in order to clarify whether proscribing their use postoperatively was a myth or had a rationale. The reason why underwire bras might be associated with an increased rupture rate is unclear. We could speculate that it might stem from repeated and consistent mechanical wear-and-tear. However, if that were the case, we would have expected more ruptures in the inferior quadrants while the opposite occurred in our study. The mechanism of rupture might indeed be caused by the reduced expansibility of the lower pole which leads to its compression and migration of pressure cranially. Nevertheless, we currently advise patients against their use in the postoperative period.

Regarding the effect of car seat belts, we would like to acknowledge that their use is a legal obligation in Italy. Individuals may be exempt from their use when they present a health condition which poses a contraindication. Those include but are not limited to at risk pregnancies, severe obesity, severe incisional hernias, severe respiratory failure, use of specific shoulder/hip braces or orthopedic corsets, certain amputations or mutilations, and the insertion of medical devices, including BIs. The legal exemption is only valid when the health condition has been certified by a physician who agrees that the condition poses a contraindication. Song et al. [41] stated in their systematic review that rupture or damage correlated to car seat belts is caused by a moderate-to-severe compression of the chest which crushes BIs between the belt and the ribcage. While it is true that seat belts might cause implant rupture, in our practice we strongly recommend patients to abide by traffic safety rules and to use them anyway. The health risks stemming from not using the seat belt are likely more concerning than the risk of BI rupture. Therefore, despite the possibility of a legal exemption, we recommend patients to use the car seat belt. In our study group, 174 patients wore the seat belts and experience 90 ruptures (51.7%), while 17 patients (8.9%) did not use them anyway, 3 of which experienced implant rupture (17.6%). Additionally, the use of seat belts was considered a dichotomic variable: patients either used them consistently or did not. It was not correlated to their profession (i.e., cab drivers) or to the number of hours spent driving, which could have been used to distinguish continuous from incidental car seat belt usage. This potentially limits the weight of our findings and deserves further investigation. In regards to diagnostic imaging, BIs are radiopaque objects which might obscure breast tissue [42]. Patients with BIs can still undergo mammography imaging using the Eklund maneuver which consists in displacing the BI pushing it back against the chest wall while pulling the breast tissue forward so it can be seen in the mammogram [43]. Implant rupture is an inherent risk of this procedure, which radiographers may inadvertently cause due to lack of guidance about when to stop compression [44]. Nevertheless, despite our findings, the benefits provided by mammographic imaging for cancer detection largely outweigh the risk of BI rupture, which is why we do not recommend discontinuation of their use.

Regarding other statistically significant factors, we found the use of heavy purses/backpacks and daily weightlifting not to increase BI rupture rate. This should be clarified since those activities affect the chest causing repeated contraction of pectoralis major muscle, which has been shown to cause elevation of the breasts and development of wrinkles or ripples in the caudal and cranial quadrants [45]. The constant muscle contraction and stress exerted by physical activity could cause an intrinsic and regular pressure on the implant, with a wear-and-tear which should result in a higher likelihood of rupture. However, no reputable evidence has currently linked repeated contraction of pectoralis major muscle with implant rupture, and neither our study does. Besides the high prevalence of submuscular implants in our population, the low number of subglandularly placed devices hinders us from being able to obtain a significant comparison. Another possible confounding factor could be that we routinely perform primary division of the thoracodorsal nerve in our implant-enhanced latissimus dorsi patients, which represent more than half of our patient population (53.9%). This approach has been shown to effectively reduce muscle contraction surrounding the BI [46]. Of note, our results are in contrast with some of the beliefs supported by other authors regarding the role of capsular contracture in the likelihood of implant rupture. In fact, Feng et al. found that grade III/IV capsular contracture is associated with a higher risk of BI rupture [38].

When a cause for BI rupture can be identified, iatrogenic damage appears to be the most frequent culprit [9, 47]. It can take many forms which include surgical instrument damage. Autologous fat transfers (AFT) represent some of these instances, where infiltration cannulas may result in the accidental BI puncture [48]. AFT is commonly performed in reconstructive patients, either as adjunct procedure to correct implant-related deformities such as waving and rippling, or to mitigate the outcomes of radiotherapy[49, 50]. Of note, out of 77 cases where AFT procedures were performed, 20 cases had implant rupture, and three were specifically found from their surgical report to have puncture sites breaching the implants’ shell with fat tissue collections inside the devices. Nevertheless, AFT and adjunct surgical procedures in general were not found to statistically increase the risk of BI rupture in our study. We believe that this might be due to certain intraoperative strategies which have been implemented to reduce the risk of accidental BI damage, such as ensuring subcutaneous visibility of the injection cannula’s tip while it is being inserted, to avoid plunging the cannula too deep.

## Conclusion

We believe that providing accurate patient information before undergoing any BI procedure is instrumental, and we believe that disregarding recommendations with no scientific backing is one major step toward that objective. In our mostly reconstructive population, patient and operative variables did not significantly influence implant survival. The same could not be said regarding the use of underwire bras, car seat belts and mammography imaging which have shown to increase the likelihood of BI rupture. Despite our findings, while we can recommend patients not to use underwire bras postoperatively, the life-saving benefits from using car seat belts and mammographic imaging cannot be dismissed, regardless of the increased rupture risk. On the other hand, several historical concerns regarding postoperative management are long outmoded, including the fact that patients should be discouraged from lifting heavy weights to reduce the risk of rupture, and we speculate that increased muscular activity does not constitute a higher risk factor. Special considerations should be mentioned for AFT procedures in implant-based reconstructions, which are safe and are not linked with a higher likelihood of rupture. Finally, our evidence confirms that implant rupture rate is directly correlated with duration of implant indwelling. Our findings aim at improving everyday clinical practice with BIs. Nevertheless a larger scale study with greater cohorts could further highlight the influence of operative variables and patient lifestyle habits on implant survival and define standard guidelines.

## Supplementary Information

Below is the link to the electronic supplementary material.

Supplementary file1 (DOCX 21 KB)
