# Supplementary material for: Assessment of Risk Factors for Rupture in Breast Reconstruction Patients with Macrotextured Breast Implants
Source: Aesthetic Plast Surg. 2022 Oct 13;47(2):517–30. doi: 10.1007/s00266-022-03118-9 (PMC10070228; doi:10.1007/s00266-022-03118-9)
Supplement: Supplementary file 1 — Supplementary file1 (DOCX 21 KB) [file 266_2022_3118_MOESM1_ESM.docx]

**BREAST IMPLANT ASSESSMENT QUESTIONNAIRE**

NAME:

SURNAME:

DATE:

1. How long have you had breast implant(s)? ........................................................................

- Year of first implantation: …………………………………………………………………………………..

1. Have you underwent any or revisional surgery after receiving you implant(s)?

- NO ………………………………………………………………………………………………………………….
- YES. If so, specify: …………………………………………………………………………………………….
- Autologous fat grafting …………………………………………………………………………..
- Scar revision ………………………………………………………………………………………….
- Nipple reconstruction ……………………………………………………………………………..
- Implant replacement ………………………………………………………………………………
- Other: …………………………………………………………………………………………………..

1. Did you receive radiotherapy? ………………………………………………………………………………………

- NO ………………………………………………………………………………………………………………….
- YES …………………………………………………………………………………………………………………

1. Have you ever received a mammography while having breast implants? …………………………..

- NO ………………………………………………………………………………………………………………….
- What other diagnostic procedures have you received? ………………………………..
- YES …………………………………………………………………………………………………………………
- When was the last time you received a mammography? ………………………………

1. Do you sleep in a prone position? …………………………………………………………………………………..

- NO ………………………………………………………………………………………………………………….
- YES …………………………………………………………………………………………………………………

1. Do you do sports (i.e. gym, jogging, etc.) at least once a week? ………………………………………..

- NO ………………………………………………………………………………………………………………….
- YES …………………………………………………………………………………………………………………

1. How many chores do you do at home? ……………………………………………………………………………

- None ……………………………………………………………………………………………………………….
- Light (under 30 minutes a day) …………………………………………………………………………..
- Moderate (30 min to an hour a day) …………………………………………………………………….
- Heavy duty (over an hour a day) …………………………………………………………………………

1. Do you lift heavy weights at home or for work (i.e. at least 5 kilograms) on a daily basis? …..

- NO ………………………………………………………………………………………………………………….
- YES …………………………………………………………………………………………………………………

1. Do you use heavy purses, backpacks or bags (i.e. over 5 kilograms) on a daily basis? …………..

- NO ………………………………………………………………………………………………………………….
- YES …………………………………………………………………………………………………………………

1. Which one of your hands is the dominant one?

- RIGHT …………………………………………………………………………………………………………….
- LEFT ……………………………………………………………………………………………………………….

1. Do you regularly use bras with an underwire? ………………………………………………………………….

- NO ………………………………………………………………………………………………………………….
- YES …………………………………………………………………………………………………………………

1. Do you use the car seat belt when driving or moving by car? ……………………………………………..

- NO ………………………………………………………………………………………………………………….
- YES …………………………………………………………………………………………………………………

1. Have you ever been in an accident, had a trauma or fall which affected the chest? ……………….

- NO ………………………………………………………………………………………………………………….
- YES …………………………………………………………………………………………………………………

1. Do you regularly massage your breasts after the receiving your implant(s)? ……………………….

- NO ………………………………………………………………………………………………………………….
- YES …………………………………………………………………………………………………………………
